# Phases 1–3 Clinical Trials Using Adult Stem Cells in Osteonecrosis and Nonunion Fractures

**DOI:** 10.4061/2010/410170

**Published:** 2010-10-26

**Authors:** Jean-Philippe Hauzeur, Valérie Gangji

**Affiliations:** ^1^Department of Rheumatology, CHU Sart Tilman, B35, 4000 Liège, Belgium; ^2^Rheumatology and Rehabilitation Medicine Department, Erasme Hospital, Free University of Brussels, 1070 Brussels, Belgium

## Abstract

Nonunion fractures and aseptic bone necrosis are two pathological conditions having some impairment of the cellular part of the repair: a reduction of MSC and of the osteoblastic activation. Both are good candidates for cell-based therapies using stem cells. We made a review of the published human trials. Only autologous bone marrow aspirate implantation was until now used. In Nonunion, a direct injection—15 to 150 ml—was made in 4 case series studies. In another, the bone marrow aspirate was concentrated before injection. The results were good. In bone necrosis, only one level 1 study was published. The results at 24 months were positive in terms of reduction of the necrosis and appearance of collapse. In 3 case series studies, a treatment with concentrated bone marrow aspirates was deemed useful with good results in 76 to 96%. These results are interesting but need confirmation by controlled studies.

## 1. Introduction

The physiological bone repair process is impaired in delayed or nonunion (NU) fractures [1] and aseptic bone necrosis (ON) [2].

Although the physiopathological factors are different, in both diseases, bone lesions are not repaired in the right time nor in the right manner.

Bone healing is produced by a cellular mechanism including mesenchymal stem cells (MSCs). The MSCs need to be recruited in the pathological area. These nonhematopoietic progenitor cells are able to be differentiated in osteoblasts under the influence of growth factors such as bone morphogenetic proteins (BMPs), platelet-derived growth factor, transforming growth factor beta, insulin-like growth factor, fibroblast growth factor, and PTH.

MSC can be found mainly in bone marrow, but also in fat tissue, synovium, periosteum, skeletal muscles, and umbilical cord. Some recent data suggest that the osteogenic differentiation capability of MSC from bone marrow and from periosteum is higher than MSC from adipose tissues [3].

Several methods could be used to increase MSC population and its osteogenic differentiation in the pathological area:

- a local injection of bone marrow aspirates,
- a preliminary culture of the bone marrow aspirate to increase the number of MSC cells,
- a preliminary culture of the bone marrow aspirate to produce an expansion and an osteogenic differentiation of the MSC,
- a genetic modification of the injected MSC to increase the secretion of growth factors like BMP and VEGF [4, 5].

In nonunion, the etiology is not clearly understood. Excessive mechanical instability of the fracture, a reduction of bone vascularity, and smoking are cited. Furthermore, some genetic predisposition could exit. In atrophic NU sites, osteoblast progenitor cells are significantly reduced [6]. In bone marrow from the iliac crest of atrophic NU bone marrow-derived mesenchymal stem cells are in smaller number and have a reduction of their proliferative capacity [7].

In nontraumatic ON, apoptosis of osteocytes and cancellous bone lining cells in the necrotic lesion and also at some distance from the lesion, in the proximal femur [8] are increased. The replicative capacities of osteoblastic cells obtained from the intertrochanteric area of the femur are reduced in patients with ON [9]. The number and the activity of fibroblast colony-forming units, reflecting the number of mesenchymal stem cells that could potentially give rise to mature osteoblasts have been shown to be decreased in ON [10, 11]. Moreover, the capillaries serving as a conduit for the stem cells and bone cells needed in bone repair in addition to providing blood supply could be altered by emboli or thrombosis in ON [12]. 

In both pathological conditions, some impairment of the cellular part of the repair could exist: a reduction of MSC and of the osteoblastic activation.

The best treatment remains to be found in both conditions. Among the different developed approaches, the cell-based therapies to improve bone repair are presented and seem to be promising. They are based on the concept of the regenerative medicine and aim to recover an optimal bone repair process.

This paper summarizes a review of the trials published in this field.

## 2. Clinical Trials in Nonunion Fractures

A recent review of the current technologies in bone-healing and repair didnot find any human study of level-I evidence concerning bone marrow aspirates, nor gene therapy [13]. Only a few studies support the therapeutic use of bone marrow transplantation in human [2].

A systematic review was conducted using Pub Med, Medline. This research was completed checking references cited in listed articles. The key words were “bone marrow”, “stem cells”, “nonunion fractures”, and “cell-based treatment”.

Unlike animals, in humans, only bone marrow (BM) aspirates implantations were until now used.

### 2.1. BM Aspirate

Connolly and coauthors should be the first to report results in a case of infected NU of the tibia [14]. In a further report of the use of marrow graft for osteogenesis from 1986 to 1995 including 100 patients having a tibial NU, a good response was found in 80% [15]. No complications were reported. The method used is made under general anesthesia. The patient was placed in a prone position and the marrow was aspirated in 3–5-ml aliquots. Simultaneously with the marrow aspiration, a second marrow needle was inserted into the site of the nonunion to directly inject the BM aspirate. The total injected volume was 100–150 ml. In 2 cases a second injection was done. No reason for this second injection was mentioned by the authors. The healing time ranged from 6 to 10 months

In 1990, Healey et al. published good results in 7/8 cases of NU after BM aspirates injection in situ [16]. These cases were all failure of osseous reconstruction (autogenous iliac crest bone grafting) after lower-extremity resections for sarcoma affecting bone. The bone marrow, 56 ml at the beginning of the series to 3 ml at the end, was aspirated from the iliac crest under general anaesthesia, and directly injected in NU, until a total of 50 ml has been grafted. No heparin was used to avoid potential impairment of bone healing associated with heparin [17, 18]. In 4 cases a second injection was made when no healing process was seen on review of serial roentgenogram. The healing time ranged from 4 to 36 weeks (mean 18).

In 1993 Garg et al. applied a technique they tested earlier in rabbits [19]. They grafted bone marrow percutaneously in 20 ununited long bone fractures (15 in the tibia, 3 in the humerus and 2 in the ulna). Under general anesthesia, 15–20 ml of bone marrow aspirates (3-4 aspirations of 5 ml) from the posterior iliac crest was directly injected into the NU sites twice, with an interval of 3 weeks. All cases were immobilized in a plaster cast. In 17/20 cases a bone fusion was observed after 5 months [6–10]. 

In 2005 Goel et al. presented results of BM grafting in tibial NU [20]. Under local anesthesia, 3–5 ml of marrow was aspirated from the anterior iliac crest and injected immediately percutaneously into and about the nonunion site. Subsequent aspirations were performed 1 cm posterior to the previous site until a maximum of 15 ml of marrow was injected. Injections were repeated at 4–6 weeks if there was no radiological evidence of callus formation. The procedure was considered a failure if there was no clinical and radiological union at 6 weeks following the third injection. The results revealed clinical and radiological bone union in 15 out of 20 patients (75%), with an average time to union following the first injection of 14 weeks. Four patients (20%) showed no evidence of union and were considered a failure. There were no cases of infection following the injection, and no complications at the donor site.

### 2.2. Concentrated BM Aspirate

Only one trial using a concentration of the BM aspirate was published. In 2005, Hernigou et al. reported the results of a retrospective study including 60 tibial NU [21]. Under general anesthesia, 300 ml BM were aspirated from both anterior iliac crests, then filtered and concentrated by centrifugation on a cell separator. The 50 ml concentrated bone marrow was injected in NU. Weight bearing was not allowed during minimum 1 month until a callus appeared. Failure was considered when no healing existed after 6 months. In 53/60 patients, bone union was obtained in mean 12 weeks (range 4–16 week). They quantified the number of injected MSC and found a significant lower count of MSC in the negative cases.

### 2.3. Other

There was until now no human study using gene modified MSC, expanded MSC or differentiated MSC in osteoblasts. Only a recent publication concerns the effect of autologous osteoblast (OB) to improve the fracture healing [22]. The autologous OB cells were obtained from a 4 weeks culture of 3–5 ml bone marrow aspirate. A mixture with 0,4 ml (12 × 10^6^ cells) and fibrin was prepared and injected under local anesthesia into the fracture area. In this randomized study, a significant fracture healing acceleration was shown.

## 3. Clinical Trials in Osteonecrosis

A systematic review was also conducted using Pub Med, Medline. This research was completed checking references cited in listed articles. The key words were “bone marrow”, “stem cells”, “osteonecrosis”, “bone necrosis”, “avascular bone necrosis”, and “cell-based treatment”.

In 2002, Hernigou and Beaujean reported the results of a noncontrolled study of femoral head osteonecrosis [23]. The patients were followed up from 5 to 11 years with a mean of 7 years. When patients were treated before collapse, hip replacement was done in 9 of the 145 hips. Total hip replacement was necessary in 25 hips among the 44 hips operated after collapse. The authors classified this study in an evidence level III. But the study didnot have any control! The evaluation was only based on a comparison with the estimated natural evolution of cases published in other studies. The correct level of evidence seems to be level IV. The method for implanting the bone marrow aspirate in the necrotic area was the same as described for the same author in NU. The volume of BM aspiration made under general anesthesia was 300 ml. A filtration and a concentration by cell separator were performed. The final volume to inject into the necrotic area was 50 ml.

In 2004, Gangji et al. published a controlled, double blind, prospective study including 18 femoral head ON before collapse treated by core decompression using a 5 mm trephine with or without concentrated BM aspirate [24]. The method to obtain and to prepare BM was the Hernigou's method. After 24 month followup, there was a significant reduction in pain and joint symptoms within the BM graft group (*P* = .021). At 24 months, five of the eight hips in the control group had deteriorated with appearance of a collapse of the femoral head, whereas only one of the ten hips in the BM graft group had progressed to this stage (*P* = .016). Survival analysis showed a significant difference in the time to collapse between the two groups. In addition, in the BM graft group, the volume of the necrotic lesion decreased by 35%.

In 2008, a publication in Chinese presented a retrospective study using another method of treatment [25]. A 3-tunnels core decompression was performed in the femoral head to allow implantation of bone marrow MSC and decalcified bone matrix. Among the 87 patients (103 hips), the average rate of excellent and good results (based on clinical and radiological evaluation) were deemed to be 75, 7% after a followup of mean 26 months. No more details were given.

In 2009, Wang et al. reported the results of 59 ON of the femoral head (before or after collapse) in a prospective noncontrolled study [26]. The 100–180 ml BM aspirate was concentrated to 30–50 ml. The implantation into the necrotic area was done through 2-3 holes made using a trocart with a 3.5 mm outer diameter. The followup was mean 27 month (range: 12–40). Clinically, the overall success was deemed in 80% and hip replacement was made in 7/59 hips (11,9%).

## 4. Discussion

In NU, this paper shows that the therapeutic effect of MSC is only supported by some studies using BM aspirate, concentrated or not, of evidence level IV. Several differences between these studies must be noted. The type of NU and the therapeutic methods were not the same. Different methods to harvest and to inject bone marrow were used. The volume and the number of injected MSC (when evaluated) were quite variable.

Good results were found in all. With small volume (15–20 ml) and without any concentration they were 83% [19] and 75% [20]. With larger volumes (300 ml) and after concentration, the good results increased slightly to 88% [21]. Clearly, the question of the best method, and the interest of larger BM aspirate volumes are not resolved. 

An additional question is the interest of an injection of large volume in lesions having a smaller volume. What is about homing and proliferation of injected MSC? Is the bone repair boosted by the injected MSC or by other components of the BM aspirate like growth factors? Trials using BMP have proven their efficacy in 7 studies with level-1 evidence [13].

In ON, the effect of BM implantation was tested in one trial level-II evidence [24] and 3 trials level-IV [23]. The method for harvesting and concentrating the bone marrow as the injected volume as the method of implantation was the same in 2 studies but used 2 or 3 tunnels core in the 2 others. Such results are very promising, but need to be confirmed in larger randomized control studies. The same answer about the relationship between the injected volume and the lesion volume needs to be studied.

Finally we do not find data to confirm that the therapeutic effect of BM aspirate should be due to its cellular part, especially MSC and not to growth factors. 

In conclusion, these reviews confirm that BM aspirate could induce bone repair in NU and ON. But the data are very preliminary and a lot of questions remain to be clarified.
